# Tibia Nailing Without C-Arm Guidance: Challenges and Successes

**DOI:** 10.7759/cureus.16797

**Published:** 2021-07-31

**Authors:** Brejesh K Prasad, Rahul Jain, Amit Kumar

**Affiliations:** 1 Orthopaedics, Employees' State Insurance Corporation Medical College, Faridabad, IND; 2 Orthopaedics, All India Institute of Medical Sciences, Raebareli, IND

**Keywords:** tibia interlocking, radiation, fracture, tak -tak technique, c arm

## Abstract

Purpose

There are increased trends in the last two to three decades to operate tibia fractures to ensure acceptable reduction over long period of time and to ensure early mobilization and return to work. This leads to frequent use of C-arm to perform the procedures. The purpose of our study is to reduce the exposure of radiation to the patient and healthcare workers during closed nailing of fractures.

Methods and materials

This is an institute-based retrospective cohort study. Patients operated with tibia interlocking nailing between November 2016 and November 2018 were shortlisted from the OT records. Seventy-six patients were shortlisted and their clinical records were retrieved. Fifty-eight patients fulfilling the inclusion criteria were included in the study. 28 patients had Tibia interlocking nailing done without using C-arm and 30 patients had tibia interlocking nailing done under C-arm guidance. IBM SPSS software was used to compare data between the groups using Chi-square test and Independent T-test with a 95% confidence interval to determine the significance.

Results

All the patients progressed to the union by six months of surgery. Average blood loss, Infection rates and time to union in both the groups were comparable. Though the average duration of surgery was significantly higher in non-C-arm group as compared to with C arm group, when the duration of individual surgeries was analysed and plotted sequentially on a chart, we found it was comparable in later cases.

Conclusions

With adequate practice, tibial nailing without C-arm is easy, requires minimal manpower, equipment and can also prove to be a lifesaver in case of equipment failure.

## Introduction

Tibia fracture is one of the common fractures in the younger age groups. Although conservative treatment of tibia fractures has stood the test of time, still more and more fractures are being managed operatively with Interlocking nails to ensure acceptable reduction over a long period of time and to ensure early mobilization and return to work [1]. Although most higher centers have the facility of C-arm in their orthopedic operating room, many centers in resource-poor country still have this deficiency either due to lack of equipment or due to lack of trained manpower to operate these equipment [2]. Many times these deficiencies force surgeons to deny operative treatment to patients with the right indication for surgery. In addition, there are increasing concerns being raised about high radiation exposure to health personnel working in such environments [3]. Most difficult and time-consuming part of nailing is distal locking. Also, risk of radiation exposure to surgeon and patient increases by 2.6 times while locking the nail distally [4,5]. This has encouraged us to devise newer ways of minimizing radiation exposure over time, like distal targeting device, SIGN (surgical implant generation network) intramedullary nailing, magnetic manual targeting device, electromagnetic navigation, handheld guides, fluoroscopy based surgical navigation, expanding self-locking nail etc. Although these methods decrease radiation exposure, special and costlier equipment or Implants are required, thus not suited for resource-poor places. Our study is to compare the technique of tibia interlocking with and without C-arm in terms of variables like average duration of surgery, average blood loss during surgery, time to union and requirement of additional operative procedures if any.

## Materials and methods

This is a retrospective cohort study. Ethical clearance for the study was taken by the ethical committee of the institute where the study was performed. All the patients operated for closed tibia fractures between November 2016 to November 2018 and fulfilling our inclusion criteria were shortlisted from the Operation Theatre (OT) records and were called telephonically for follow-up with all their records. The inclusion criteria were all patients aged between 18 to 50 years having isolated closed tibial shaft fractures AO OTA - 42 A1 to 42 B2. All those patients who presented one week after injury, having comorbid conditions like diabetes, established osteoporosis and having other associated systemic injuries were excluded from the study. Out of 76 patients contacted, 65 patients were able to visit timely for follow-up. Out of these 65 patients, five were excluded due to incomplete records and two were excluded due to minor head injuries at the time of index surgery. Their OT records and pre-operative X-rays were assessed retrospectively. Patients were sorted into two groups, those operated without C arm (group A) consisting of 28 patients and those operated under C arm guidance (group B) consisting of 30 patients. Each fracture was classified according to AO classification of tibial shaft fracture. Duration of surgery and intra-operative blood loss was taken from anaesthesia records, immediate post-operative X-rays were assessed for adequacy of reduction and follow-up X-rays at 3, 6, 12 weeks and at six months was assessed for union using RUST (Radiographic Union Scale in Tibia) scores [6]. The data were collected and analysed using SPSS software for statistical significance using chi-square test, chi-square test with Yates correction and Independent T-test for statistically significant difference at a confidence interval of 95%.

Surgical technique

**Figure 1:**
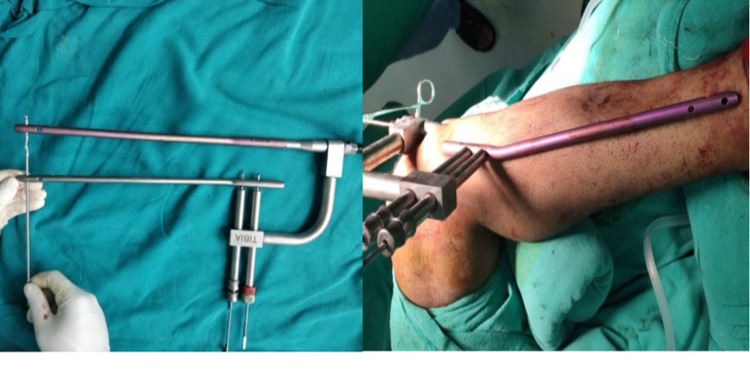
Picture showing the zig-nail-guiding nail assembly both on table (left) and in situ (right).

For the case operated without C-arm, the alignment in saggital plane was confirmed by palpating the anterior border of tibia and alignment in coronal plane and rotational alignment was confirmed with the help of an imaginary plum line dropped from tibial tuberosity to the 2nd metatarsal. With the knee flexed 90 degree overhanging from the edge of table entry was made with a bone awl, through the patellar tendon splitting approach. The entry point is made in the line of medullary canal i.e. 3 mm medial of the tibial crest in the frontal plane and just distal to the angle between the tibial plateau and anterior tibial metaphysis in the saggital plane. Advancing of bone awl into medullary cavity is confirmed by the oozing fatty marrow. Guidewire was passed into the proximal fragment and negotiated across the fracture site after alignment of the fracture ends. Passage of guidewire into the distal fragment was confirmed by the grating sensation and a rigid endpoint when it hit against the distal plafond. Endpoint was also confirmed by the length of guidewire passed which should be approximately equal to preoperatively measured tibial tuberosity to medial malleolus distance (TMD). Reaming was done 1.5mm higher to the nail diameter to be inserted. The endpoint of reaming was confirmed by chattering sensation as reamer comes in contact with internal cortical surfaces. Adequate sized nail was loaded over zig was passed along the guidewire. Limb rotational alignment was confirmed by tibial tuberosity, tibial shin and 2nd metatarsal line. Distal locking was done in figure of 4 position. Distal holes were localized by putting a similar length nail over the leg and matching the proximal locking zig to both the proximal holes of the external nail and secured into position by drilling the outer cortex only, through the zig & drill sleeve (Figure 1). Engaging both the proximal holes of the outer nail is important to prevent anteroposterior translation of distal holes. Guide was passed through the intramedullary nail and the length at which a rigid endpoint reached was marked with a guidewire holder or a Kocher’s forceps. After this, the guidewire was partially withdrawn without changing the position of the Kocher’s. Near cortex of the distal most hole was drilled with a 4.9 mm drill bit and on getting a give-away, the guidewire was again pushed in to check the new level of rigid endpoint achieved, if the Kocher's forces are seen more withdrawn than the previous endpoint, it indicates that the drill bit is going through the nail and that we are on the right track. If the drill bit is on a wrong track and the trajectory needs to be changed, it should be attempted with a 2.0 mm k wire and again confirmed by sounding with guidewire. Using a k wire, the right trajectory is confirmed by a rigid endpoint when the guidewire hits the K wire, as well as slight movement of the k wire when it gets hit by the guidewire. After the trajectory was confirmed and both cortices drilled, the distal locking was completed with adequately sized locking bolts. The correct placement of locking bolts also can be confirmed by guidewire. Proximal locking was completed with zig guidance. First 34 patients were operated without C-arm, due to its non-availability at that time and all the necessary information was recorded in the record sheets. After the availability of C-arm in our institute, all the patients were operated under C arm guidance with freehand locking technique and same information was recorded in the case records.

## Results

**Figure 2:**
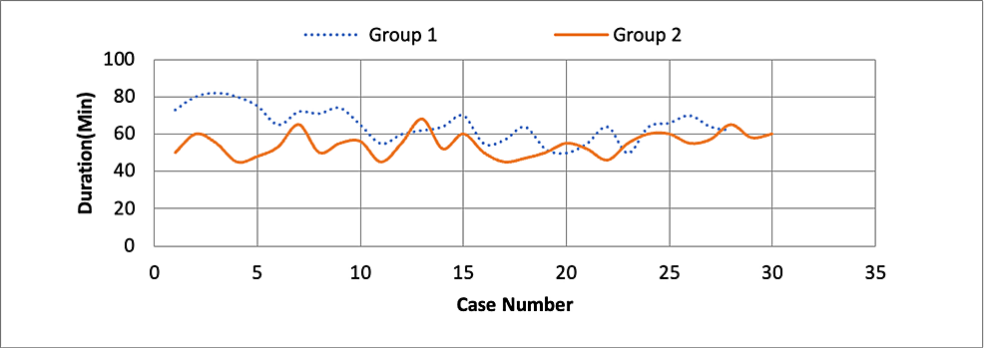
Duration of surgery plotted against serial number of case in each group.

As shown in Table 1, Group A had 28 patients of which 16 were males and 12 females, Group B had a total of 30 patients of which 16 were males and 14 females. The mean age of patients in group A was 37.18 years and that in group B was 37.87, the age difference and sex distribution between the two groups was statistically insignificant. The mean duration since injury in group A was 2.61 days while in group B it was 2.80 days, the difference in days since trauma was not clinically significant. The duration of surgery from skin incision to completion of last suture was calculated using the OT notes and the nursing chart. In group A the mean duration of surgery was 65.04 minutes while in group B it was 54.40 minutes, this difference in duration of surgery was clinically significant and suggests that significantly more time was consumed when distal locking was done without using C-arm using nail over nail technique. When we plotted the time duration of each surgery on a chart (Figure 2) we could clearly see a decrease in time duration as the number of cases progressed in group A whereas for group B it remained almost static, thus pointing to faster surgery as the team got used to the steps of surgery.

**Table 1:** Demographics and other data.

		Group A (n = 28)	Group B(n = 30)	p-value
Age (mean)		37.18 years	37.87 years	>0.05
Sex	Male	16	16	>0.05
	Female	12	14	
Mean duration since injury to treatment		2.61 days (SD±1.03)	2.80 days (SD± 0.92)	>0.05
Mean duration of surgery		65.04 minutes (SD±8.92)	56.40 minutes (SD±6.19)	<0.05
Mean blood loss during surgery		145.6 ml (SD±15.94)	143.1 ml (SD±10.97)	>0.05
Need for other procedures	Dynamization	4 (14.28%)	1 (3.33%)	>0.05
	Bone grafting	2 (7.14%)	4 (13.33%)	
	Nailing	1(3.57%)	Nil	
Number of patients showing union at 6 months		23 (82.14%)	25 (83.33%)	>0.05

## Discussion

Although various levels of radiation dose have been deemed to be safe for personnel working in radiation industries, the irony is that no safe threshold level of radiation exists. Studies have shown an increased risk of cancer as high as seven times among orthopedic surgeons as compared to general population and five times as compared to other doctors [7]. Mustafa et al. showed that median number of fluoroscopy per tibia interlocking nail to be 202 which carried radiation dose with median dose area product (DAP) of 359.5 mGy per square centimeters [3]. Considering the increased relative risk of various cancers in operating room personnel, various methods are being explored and devised to reduce the radiation exposures during surgeries. These include measures to assess reduction using bony landmarks or using alternative tricks to confirm and guide distal locking in tibia and femur fractures. For distal screw locking freehand technique is the most commonly used method. Knudson et al used K-wire whereas Macmillan used Steinman pin [8,9]. Various methods have evolved over time to decrease this radiation exposure to the extremities of operating surgeons. Hashemi et al used handheld jigs, Rao used forceps to place a metallic washer to locate perfect circle [10,11]. various image intensifier mounted device have also been used by Kempf et al and Tyropoulous and Garnavos [12,13]. Due to the unavailability of C-arm, Kanellopaulos et al. locked under direct vision using a 3-4 cm cortical window [14]. Various nail-mounted jigs have been used but these guides failed often as it could not account for intramedullary bending of nail [15].

We assessed the reduction with the help of bony landmarks in tibia fractures, used nail over nail technique for guiding distal locking and used the Tak-tak technique for confirming the right placement of distal screws. Using these radiation-avoiding techniques against our normal technique of tibia interlocking nailing, we assessed the groups for adequacy of reduction, blood loss during surgery, duration of surgery, and progress of fracture healing. The free hand technique is chosen for comparison because it remains the predominant method used by orthopaedic surgeons for distal locking. Using the nail over nail technique we were able to complete the nailing procedure in the 28 patients of Group A in 65.04 minutes which is significantly higher than Group B (54.40 minutes) (p-value < 0.05). Thus it is evident that in our study the time required to perform tibia interlocking nailing was significantly higher without C-arm as compared to with C arm. This was in contrast to previously published study by R Rohilla et al in which the operating times between the two groups was not statistically significant [16]. Looking back at our data and plotting the same a graphical interface revealed that the case durations had gradually come down from the 1st to the 28th case, thus hoping us to believe that as our experience and confidence increased, the duration of surgery gradually decreased.

This technique does not require any additional equipment, instrument or manpower to practice. The external nail used as a guide can be reused after sterilization, and since every time a fresh nail is used as a guide, there is no loss of accuracy due to deformation as in distal zig after repeated use. To avoid forcible hammering and deformation, the diameter of nail used is 0.5 to 1.5 mm less than the maximum sized reamer used. Over reaming did not affect union as evident from the comparable RUST scores at six months in both the groups in our series.

## Conclusions

Repeated concerns have been raised regarding the over-dependence on C-arm leading to various techniques being described and published which require nil or lesser radiation exposure for interlocking nailing. Our technique is an amalgamation of various techniques described to achieve and maintain adequate reduction and achieve distal locking without using any additional equipment. Our study also emphasizes the need to practice this technique so as to be useful in routine and emergency situations without adversely affecting patient outcomes.
